# Treatment Outcome of Carcinoma Vulva Ten-Year Experience from a Tertiary Cancer Centre in South India

**DOI:** 10.1155/2017/7161437

**Published:** 2017-12-14

**Authors:** Sakthiushadevi Jeevarajan, Amudhan Duraipandian, Rajkumar Kottayasamy Seenivasagam, Subbiah Shanmugam, Rajaraman Ramamurthy

**Affiliations:** ^1^Department of Surgical Oncology, Tamil Nadu Multi-Super Specialty Hospital, Chennai, Tamil Nadu, India; ^2^Department of Surgical Oncology, Thoothukudi Medical College, Thoothukudi, Tamil Nadu, India; ^3^Lancashire Teaching Hospitals NHS Foundation Trust, Royal Preston Hospital, Preston, UK; ^4^Department of Surgical Oncology, Coimbatore Medical College & Hospital, Coimbatore, Tamil Nadu, India; ^5^Department of Surgical Oncology, Government Royapettah Hospital and Kilpauk Medical College, Chennai, India

## Abstract

**Background:**

Carcinoma vulva is a rare disease accounting for 1.3% of all gynaecological malignancies. The present study is a 10-year retrospective review of our experience of the surgical options, morbidity, failure pattern, and survival for invasive carcinoma vulva.

**Materials and Methods:**

Retrospective analysis of case records of 39 patients who underwent surgery for invasive vulval cancer between 2004 and 2013 in the Department of Surgical Oncology at the Government Royapettah Hospital, Chennai.

**Results:**

The median age was 55 years. Radical vulvectomy was the preferred surgery. 31 patients underwent lymphadenectomy. Seroma formation and groin skin necrosis were the most common postoperative complications. With a median follow-up of 32 months, 8 patients (20.5%) developed recurrence (systemic = 1, regional = 4, and local = 3). The estimated 5-year disease-free survival (DFS) was 65.4% and the overall survival (OS) was 85.1%. On univariate analysis, stage and lymph node involvement significantly affected OS. Nodal involvement with extracapsular spread (ECS) significantly affected both DFS and OS.

**Conclusion:**

The treatment of carcinoma vulva should be individualized with multidisciplinary cooperation. The paucity of data, especially from India, necessitates the need for more studies, preferably multicentric, keeping in mind the low prevalence.

## 1. Introduction

Carcinoma vulva is relatively a rare disease accounting for 1.3% of all gynaecological malignancies [1] and 0.3% of all cancers affecting females in India [2]. Predominantly it is the disease of elderly women with the median age being 67 years, although it is now becoming common in younger age groups [3]. Most patients in developing countries like India present with advanced locoregional disease for various socioeconomic reasons including lack of awareness resulting in poorer outcomes and posing management challenges [4].

There is a striking paucity of literature about carcinoma vulva from developing countries including India. Due to its rarity, large prospective randomized trials that can guide management are few. The purpose of this study is to know the demographic pattern of invasive vulvar cancer, to analyse the surgical options, the postoperative complications, failure pattern, and survival following surgical management and to compare our results with other published series.

## 2. Materials and Methods

Patients who underwent surgery for invasive vulvar cancer between the periods 2004 to 2013 in the Department of Surgical Oncology at the Government Royapettah Hospital were analysed. The data was collected from clinical and follow-up records. All patients were treated with curative intent, and followed up regularly as per protocol. Preoperative evaluation consisted of clinical examination, routine blood and urine tests, chest radiography, and contrast enhanced computed tomography (CECT) of abdomen and pelvis including the groin. Selected patients were additionally evaluated with examination under anaesthesia (EUA), MRI pelvis, cystoscopy, and proctoscopy. Histopathological documentation of the primary lesion was done preoperatively. Due to financial constraints, human papillomavirus (HPV) status was also not evaluated.

Surgical management options included radical vulvectomy (RV), simple vulvectomy (SV), hemivulvectomy (HV), and wide local excisions (WLE) with or without unilateral/bilateral inguinofemoral node dissections and iliac node dissections as required.

In our centre, RV was done using three separate incisions (Figures 5 and 6), one for primary tumour and one for each groin dissection. Primary tumour was removed with a minimum of 1 cm margin in all directions with the incision extending down to the inferior fascia of the urogenital diaphragm. Both labia majora and minora including the clitoris were removed along with the tumour. In WLE, the primary tumour was resected with a minimum of 1 cm margin in all directions, with the incision extending down to the inferior fascia of the urogenital diaphragm, preserving the other uninvolved part of vulva. In simple Vulvectomy (Figure 3), the skin, subcutaneous tissue, labia majora, labia minora, and clitoris were removed en bloc with the tumour, but it does not require an incision all the way to the perineal fascia like RV.

Inguinofemoral block dissection (IFBD) (Figure 4) either unilateral or bilateral was performed through transverse incisions below the inguinal ligament. Usually sartorius transposition was done. The practice of saphenous vein preservation was not followed. Earlier in our institution, iliac node dissection was done if the inguinal nodes were positive by frozen section, but very soon that practice was abandoned.

Adjuvant external beam radiotherapy was given with 50 Gy in 2 Gy per fraction, 2D, nonconformally, once daily 5 days a week, for 5 weeks, if indicated (margin positivity, involvement of more than 1 node, and presence of extracapsular nodal extension irrespective of the number of nodes) as per MDT decision.

Patients were followed up monthly in the first year, twice monthly in the second year, three times monthly in the third year, six times monthly for the fourth and fifth years, and yearly thereafter. Follow-up included clinical examination at each visit, yearly chest X-ray and CECT abdomen with pelvis, and other investigations as indicated.

The demographic pattern, management options, postoperative complications, failure pattern, and survival were analysed. Survival analysis was done using Kaplan-Meier method with SPSS 17 (SPSS Inc, USA) for Windows Software. The important prognostic variables were analysed using log-rank test in the univariate analysis. *P*-value of less than 0.05 was considered as statistically significant. Results were compared with other published data.

## 3. Results

During this 10-year period, 39 patients with invasive vulvar carcinoma were treated with surgery.

The mean and median ages of the patients were 52.5 and 55 years, respectively (range: 23–73 years). Of those 39 patients, 62.9% were below 60 years of age. Thirty-six (92.3%) patients presented with ulcers over external genitalia, 30 (76.9%) with pruritus, 12 (30.7%) with pain, and 6 (15.3%) with other complaints (like discharge, swelling). Labia majora was the predominant site of disease in 80%, labia minora in 14.3%, and clitoris in 5.7%.

The various surgical procedures including lymph node dissections are as listed in Table 1. Overall, 8 of 39 patients did not have any lymphadenectomy. Of these, one patient who had initially presented with multiple verrucous lesions and underwent simple vulvectomy subsequently developed nodal recurrence at 5 months and was treated with right ilioinguinal block dissection (IIBD) followed by adjuvant RT. The remaining 7 patients had well lateralized disease with stromal invasion < 1 mm and hence nodal dissection was not done.

In 3 patients with well lateralized lesions, unilateral IFBD with hemivulvectomy or wide local excision was done. Four other patients who underwent simple vulvectomy had unilateral lymphadenectomy since contralateral nodes were negative on frozen section.

All patients had squamous cell carcinoma (SCC). All had pathological R0 resections except 3 (2 positive margins, 1 close margin). Following nodal dissection, 45.2% (14/31) were found to be node positive and 54.8% (17/31) negative. Three of the node negatives and 4 of the node positives patients developed recurrence. A total of 4 patients had extracapsular nodal spread (ECS), of which 3 died and 1 defaulted follow-up. In our series, the median node retrieval was 8 which is above the recommended number of 6 nodes [5]. Patients were staged according to FIGO-2009 staging system (stage I-20, II-4, III-13, and IV-2 patients) [6].

We had no perioperative (30-day) mortality. Postoperative complications were mainly due to nodal dissection (Table 2). Seroma was the most common complication requiring repeated aspirations in 58.1% (18/31). Skin flap necrosis (45.2%) with wound gaping was the troublesome complication following nodal dissection. It was salvaged with the removal of the necrosed part followed by secondary suturing in some cases, split skin graft in few cases. A total of 5 patients required flap reconstruction of wound (one for postvulvectomy area, four for inguinal wound), of which 2 had flap necrosis. One patient developed deep venous thrombosis (DVT) in the immediate postoperative period which was managed successfully. The majority of patients developed some amount of lower limb lymphedema following nodal dissection but not to the extent of producing significant symptoms.

Twelve patients were candidates for adjuvant radiotherapy, of which 5 declined therapy. Of those 5 defaulters, 2 with nodal positivity remained disease-free for 36 & 41 months, respectively, One with margin positivity and negative nodes was disease-free at 67 months. One with extracapsular nodal disease, developed regional recurrence at 7 months and the other patient retroviral positivity who underwent radical local excision remained disease-free till last follow-up (39 months).

At a median follow-up of 32 months (range: 2–67 months), 8 (22.9%) developed recurrence, one systemic, 4 regional, and 3 local. An interesting observation is that all regional and systemic recurrences occurred within 1 year and all local recurrences occurred after 1 year of primary surgery.

Of the 4 patients with regional recurrence, 2 had nodal and 2 had soft tissue recurrence. Of the 2 nodal recurrences, the one with mobile node was treated with right IIBD followed by adjuvant RT. The other one with fixed nodal mass had poor response to RT and chemotherapy and died. Of the two soft tissue recurrences, one developed an ulcer in the left inguinal region 6 months after the primary surgery and received RT but succumbed to the disease 4 months later. The other one developed recurrence at 3 months and was salvaged with chemoradiation and is on regular follow-up to date without disease.

Of the 3 patients with local recurrences, 1 developed recurrence 33 months after surgery. She was planned for RT, but defaulted therapy. One developed recurrence 21 months after primary surgery and RT, was planned for chemotherapy but could not complete the course, and died after 4 months. The third developed periurethral recurrence 21 months after primary surgery and defaulted therapy. Only one patient developed systemic metastases involving lung and humerus and was treated with local RT to bone and systemic chemotherapy with CDDP & 5FU.

**Figure 1:**
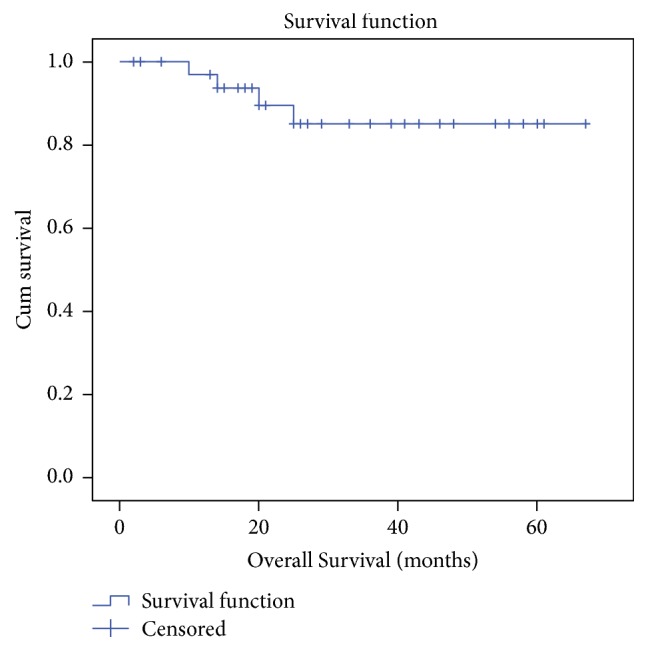
Kaplan-meier curve-overall survival for all cases.

**Figure 2:**
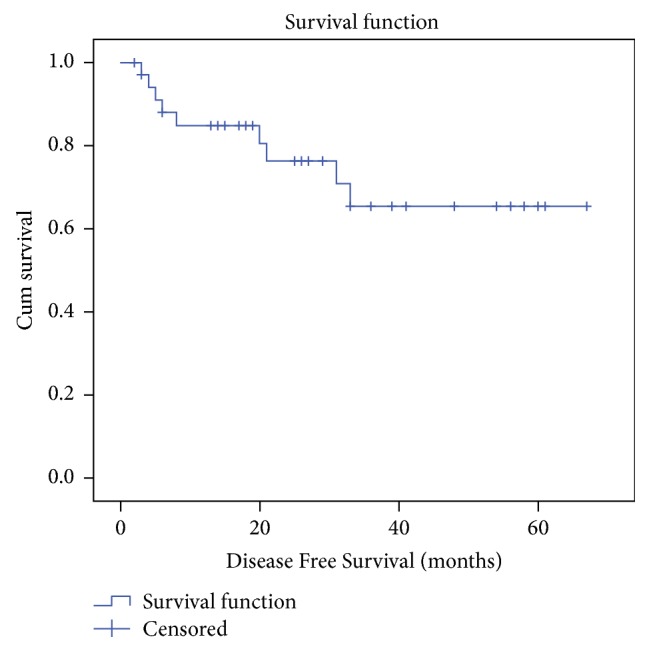
Kaplan-meier curve-disease-free survival for all cases.

**Figure 3:**
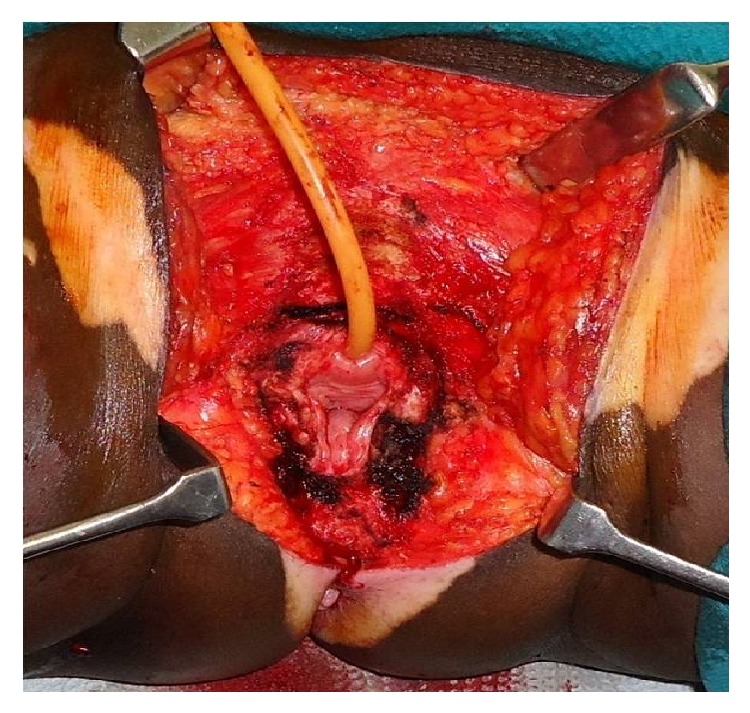
Simple vulvectomy.

**Figure 4:**
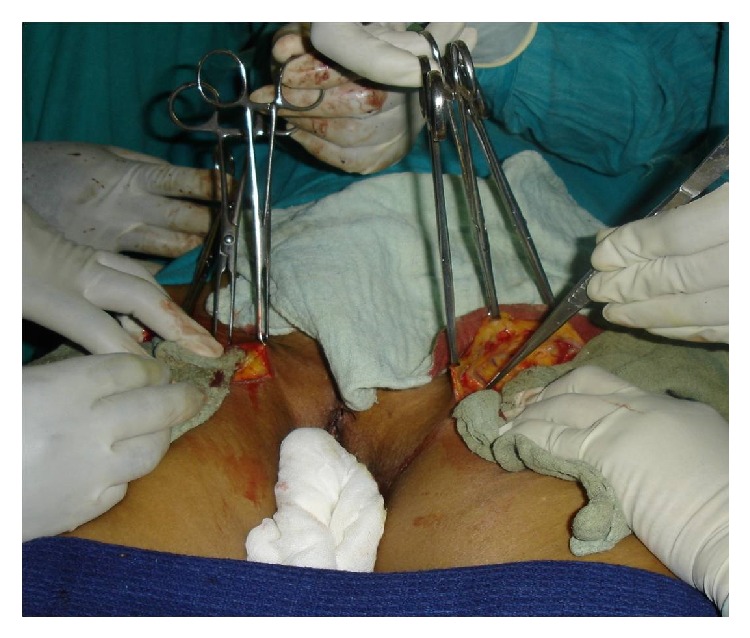
Inguinofemoral block dissection.

**Figure 5:**
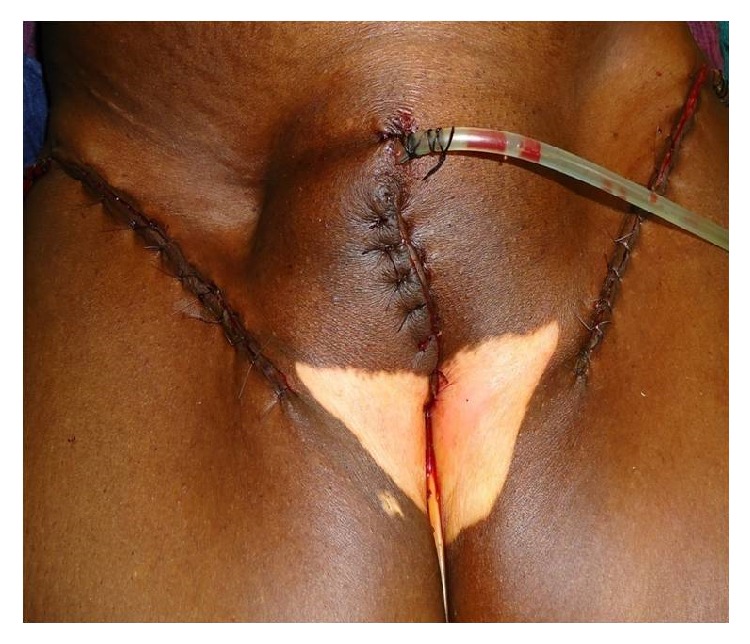
After wound closure.

**Figure 6:**
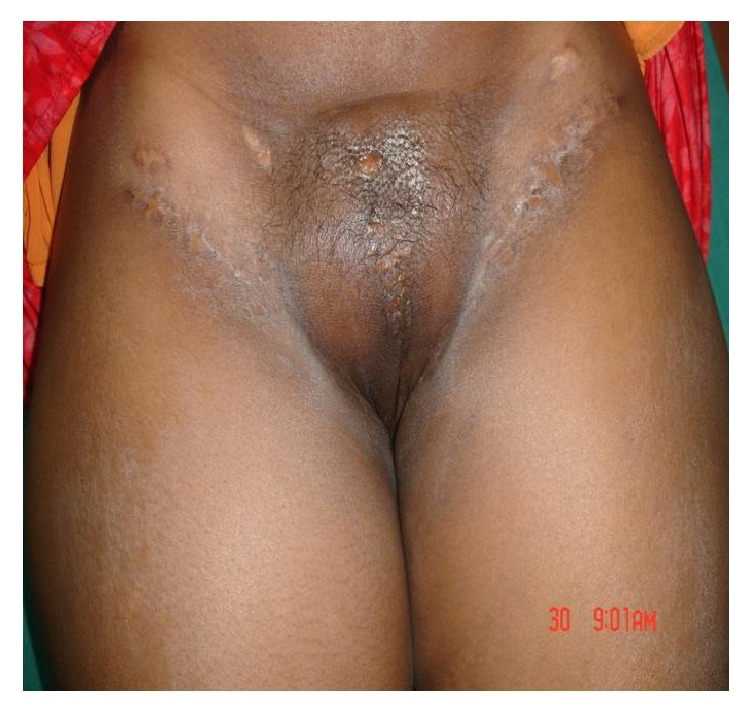
Follow-up picture.

Of those 39 patients, 5 (12.8%) died and 6 (15.3%) defaulted follow-up. Of the remaining 28 patients, 4 were alive with disease and 24 were on regular follow-up without disease. The estimated 5-year OS and DFS for all cases using Kaplan-Meier analysis were 85.1% and 65.4%, respectively (Figures 1 and 2). On univariate analysis, using log-rank test, stage, and nodal positivity were identified as significant prognostic factors for OS. An important observation was that the estimated 5 year OS for patients with extracapsular nodal spread (ECS) was significantly less when compared to nodal positivity without ECS (*P* = 0.02) and node negativity (*P* < 0.001). Given the small number of cases multivariate analysis was not possible. In our series, extracapsular nodal spread (ECS) was the only factor that significantly affected both DFS and OS on univariate analysis. The DFS for patients with ECS was significantly less when compared to nodal positivity without ECS (*P* = 0.02) and node negativity (*P* = 0.01)

## 4. Discussion

Carcinoma vulva is a rare cancer, mainly affecting elderly women. Our patients were younger when compared to other published series (Table 3). Labia majora was the most common site of disease in our series (80%) followed by labia minora (14.3%) and clitoris (5.7%). Hampl et al. in their series of 224 patients reported that the tumour localization changed significantly from the labia to the area between the clitoris and the urethra (38.4%) [3]. Given the small number in our series, such a shift in location could not be commented on.

Radical surgery still has its place even in this era of organ conservation. Most of our surgeries were radical vulvectomies and most had some form of lymph node surgery. After the landmark study by Hacker et al. the traditional radical en bloc resection of the vulva and inguinofemoral nodes through single incision underwent a drastic change in favour of separate vulvar and groin incisions which achieved similar cure rates with less morbidity [15]. After the study by Homesley et al. [16] routine pelvic lymphadenectomy was replaced with postoperative radiotherapy.

Studying the relationship between surgical margins and local recurrence, Heaps et al. reported no failures in 91 patients whose closest tumour margins were 8 mm or more in the fixed specimen [17]. However, in a study by Groenen et al. [14] the local recurrence rate did not differ between patients in whom the margin was <8 mm or 8 mm and above. We follow the practice of giving at least 1 cm gross margin.

Homesley et al. in a series reported that 24% of patients with clinically negative nodes had positive nodes on pathology and 24% the other way around, suggesting that clinical examination is inadequate in assessing the nodal spread [18]. In our series, 36.4% of patients with clinically suspicious nodes had negative nodes and 35% with clinically negative nodes had positive nodes on pathology supporting his view. In a series by Le et al. the total number of nodes harvested during surgery was proved to be an independent predictor of both progression-free and overall survivals. They propose to define optimal inguinal nodal dissection using a cut-off value of at least 10 nodes in total for bilateral IFBD [7]. In our series the median nodal yield for unilateral and bilateral IFBD were 8 and 17, which indicates optimal dissection.

The major morbidity of vulvar cancer surgery follows lymphadenectomy. Recently, a number of investigators explored the use of intraoperative lymphatic mapping to identify sentinel node that would predict the presence or absence of regional metastases [19]. Participants in a 2008 expert panel at an International Sentinel Node Society Meeting concluded that sentinel node biopsy “is a reasonable alternative to complete inguinal lymphadenectomy when performedby a skilled multi-disciplinary team in well selected patients” [20]. We are yet to practice sentinel node biopsy in vulvar cancer.

In our series 45.2% (14/31) of the patients developed skin flap necrosis and in other Indian series 88.4% had considerable groin wound dehiscence following lymphadenectomy. Comparing saphenous vein sparing to saphenous vein ligation during inguinal lymphadenectomy, Zhang et al. reported significant decrease in the development of short-term lower extremity lymphedema and phlebitis but not seroma and acute cellulitis in the saphenous vein spared group [21]. However Soliman et al. conclude that “wound complications after inguinofemoral lymphadenectomy are very high, with no single pre, intra, or postoperative factor that could be incriminated, and saphenous vein sparing provided no significant difference in decreasing local complications” [10]. In our institution we do not practice saphenous vein sparing. Judson et al. reported that sartorius muscle transposition was not beneficial based on a randomized controlled trial [22]. However, we routinely practice this technique because, in situations where inguinal wound gaping occurs due to skin flap necrosis this will prevent exposure of major vessels.

In our series, 5 (14.3%) patients defaulted follow-up. However, poor follow-up in Indian females due to several factors like long travelling distance, poor socioeconomic status, and elderly age is not unusual [8]. In our series, the recurrence rate was comparable to other series (Table 4).

Because of the changes in FIGO staging system over a period of time, stage by stage comparison of survival between series may not be representative. In our series, stage, lymph node positivity, and nodal positivity with ECS significantly affected OS on univariate analysis. FIGO stage and node positivity were the two significant prognostic factors for survival also in other Indian series [19]. Many series identified nodal status as the most significant prognostic factor among all tumour-related variables and proposed that certain variables related to positive nodes (such as ECS) could be critical for further risk assessment [23]. In contrast to these results, nodal positivity did not retain its prognostic significance in few studies [24, 25]. In our series nodal positivity with ECS significantly affected the OS and DFS like others [26–28].

The estimated 5-year DFS and OS in our series were 65.4% and 85.1%, respectively. The 5-year OS was 75.9% in a series from Singapore [29] and 41% from an Indian series [8]. In our study, all regional and systemic recurrences occurred within 1 year and all local recurrences occurred after 1 year of primary surgery.

## 5. Conclusion

In carcinoma vulva, treatment should be individualized with multidisciplinary cooperation. Stage, nodal positivity, and nodal positivity with ECS were significant prognostic factors for survival on univariate analysis in our series. The paucity of data, especially from India and other developing countries, necessitates that we urge the need for more studies preferably multicentric, keeping in mind the low prevalence. Uniform consensus should be derived from those studies regarding organ conservation strategies and morbidity reducing approaches.

## Figures and Tables

**Table 1:** Surgical procedures.

Surgery for primary tumour	Number of patients	Node dissection		
		Unilateral	Bilateral	None
Radical vulvectomy	28	0	28	0
Simple vulvectomy	4	0	0	4
Hemi vulvectomy	2	2	0	0
Wide local excision	5	1	0	4

Total	39	3	28	8

**Table 2:** Postoperative complications.

Complication	Frequency	Percentage %
Seromas requiring serial aspiration	18/31	58.1
Flap necrosis	14/31	45.2
Wound infection	2/39	5.12
Deep vein thrombosis	1/39	2.56

**Table 3:** Mean and Median age of the patients in different series.

Study	Study period	Total number of patients	Mean age in years	Median age in years	Age range in years
Bafna et al. [4]	1996–2000	37	54.7	60	24–80
Le et al. [7]	1980–2004	58	-	71.3	28.3–90.9
Sharma et al. [8]	1998–2005	60	-	63	24–92
Hampl et al. [3]	1998–2007	102	57	-	18–93
Eke et al. [9]	1998–2009	11	61.2		54–79
Soliman et al. [10]	2002–2009	34	-	56.5	23–86
*Present series*	*2004–2013*	*39*	*52.5*	*55*	*23–73*

**Table 4:** Recurrence in various series.

Series	Study period	Total number of patients	Median follow-up in months	Recurrence in percentage
Bafna et al. [4]	1996–2000	37	-	32.4
Cheng et al. [11]	1980–2002	100	-	34
Woelber et al. [12]	1996–2003	103	36	13.6
Le et al. [7]	1980–2004	58	37	29.3
Landrum et al. [13]	1990–2005	175	54.5	13
Sharma et al. [8]	1998–2005	60	23	43
Groenen et al. [14]	2000–2005	93	31	23
*Present series*	*2004–2013*	*39*	*32*	*20.5*
